# Methodological Appraisal of Phase 3 Clinical Trials in Geographic Atrophy

**DOI:** 10.3390/biomedicines11061548

**Published:** 2023-05-26

**Authors:** Marc Biarnés, Xavier Garrell-Salat, Alba Gómez-Benlloch, Mercè Guarro, Gabriel Londoño, Elena López, Sergi Ruiz, Meritxell Vázquez, Laura Sararols

**Affiliations:** 1 OMIQ Research, Carrer de Pedro i Pons 1, 08195 Sant Cugat del Vallès, Spain; xgarrell@omiq.es (X.G.-S.); agomez@omiq.es (A.G.-B.); mguarro@omiq.es (M.G.); glondono@omiq.es (G.L.); elopez@omiq.es (E.L.); sruiz@omiq.es (S.R.); mvazquez@omiq.es (M.V.); lsararols@omiq.es (L.S.); 2 Department of Ophthalmology, Hospital General de Granollers, Av. Francesc Ribas s/n, 08402 Granollers, Spain

**Keywords:** age-related macular degeneration, clinical trials, endpoints, geographic atrophy, statistics

## Abstract

Geographic atrophy (GA) secondary to age-related macular degeneration is a common cause of blindness worldwide. Given the recent approval of the first therapy for GA, pegcetacoplan, we critically appraise methodological aspects of the phase 3 clinical trials published so far in this disease in relation to their design, analysis and interpretation. We reviewed some of the key attributes of all phase 3 clinical trials in GA available in the main public registry of clinical trials as of 20 May 2023. The topics discussed included types of endpoints, eligibility criteria, *p*-value and effect size, study power and sample size, the intention to treat principle, missing data, consistency of results, efficacy–safety balance and application of results. Five phase 3 clinical trials have reported results, either partially or completely: GATHER1, DERBY/OAKS, CHROMA/SPECTRI, SEATTLE and GATE. Although there are many similarities between these trials in terms of endpoints or broad eligibility criteria, they differ in several aspects (metric of the primary endpoint, sample size, type of adverse events, etc.) that can influence the results, which are discussed. Readers should understand key methodological aspects of clinical trials to improve their interpretation. On the other hand, authors should adhere to clinical trial reporting guidelines to communicate what was done and how it was done.

## 1. Introduction

**Figure 1:**
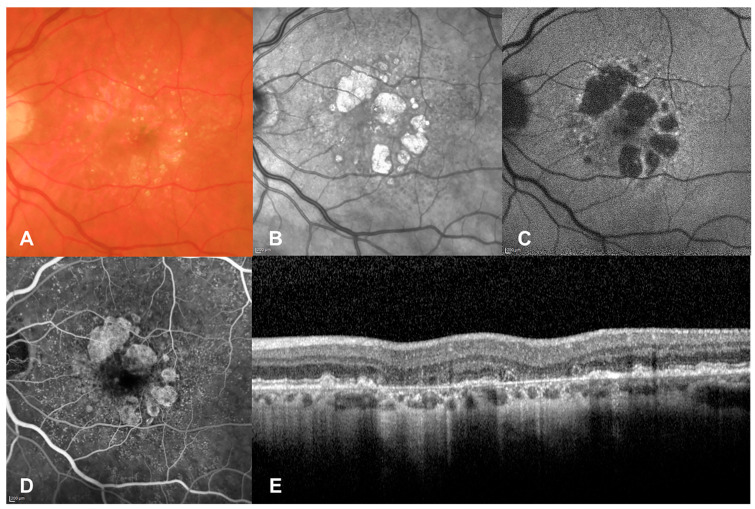
Geographic atrophy on multimodal imaging. (**A**) color fundus photography; (**B**) infrared; (**C**) fundus autofluorescence; (**D**) fluorescein angiography; (**E**) extrafoveal spectral domain optical coherence tomography. The perifoveal, multifocal areas of RPE atrophy are hyperreflective in (**B**), hypoautofluorescent in (**C**), seen as window defects in (**D**), and as RPE and outer nuclear layer loss with secondary retinal thinning and choroidal hypertransmission in (**E**). Scattered drusen are also present. RPE: retinal pigment epithelium.

Geographic atrophy (GA) is the advanced form of dry age-related macular degeneration (AMD; Figure 1). It is characterized by the progressive loss of photoreceptors, retinal pigment epithelium (RPE) and choriocapillaris [1]. As such, areas affected by GA correspond to absolute scotomata [2], which will affect best-corrected visual acuity (BCVA) if the fovea is involved [3]. Nonetheless, even if atrophy surrounds it (the so-called foveal sparing), patients experience difficulties in reading, driving and recognizing faces, which impairs their quality of life [4,5]. The estimated worldwide prevalence of GA is 5 million, which will probably grow in the next decades due to increases in life expectancy. No treatment was available for this condition until 17 February 2023, when Apellis Pharmaceuticals announced that the Food and Drug Administration (FDA) had approved Syfovre^®^ (intravitreal pegcetacoplan 15 mg) based on a slower progression of atrophy in the DERBY and OAKS phase 3 randomized controlled clinical trials.

We reviewed published phase 2/3 and 3 clinical trials in GA to highlight some aspects of their design, analysis, and interpretation (Table 1). Phase 1 and 2 trials have specific aims (dose finding, study of pharmacokinetics, etc.), and will not be addressed here. The purpose of this manuscript is to critically appraise key issues in clinical trials to aid in their interpretation using examples from the field of GA.

### Phase 3 Clinical Trials in Geographic Atrophy

There have been many trials on GA conducted for the past 15 years, but only a few reached phase 3 and have been posted in www.clinicaltrials.gov (accessed on 11 April 2023; Table 2). Only those with available complete or preliminary results released on the primary endpoint will be discussed: GATHER1 (NCT02686658) [6], DERBY (NCT03525600) and OAKS (NCT03525613, the preliminary results of which have been reported in different Meetings between 2021 and 2023 and are available at the Apellis website [7]), CHROMA (NCT02247479) and SPECTRI (NCT02247531) [8], SEATTLE (NCT01802866) [9] and GATE (NCT00890097) [10]. 

Briefly, in GATHER1, intravitreal injections of 2 mg or 4 mg avacincaptad pegol (Zimura^®^, IVERIC Bio Inc., Cranbury, NJ, USA), a C5 inhibitor, were administered monthly [6]. In DERBY/OAKS, pegcetacoplan 15 mg (Syfovre^®^, Apellis Pharmaceuticals, Waltham, MA, USA), a C3 inhibitor upstream of C5, was administered monthly or every other month (EOM) by intravitreal injections [7]. CHROMA and SPECTRI used 10 mg lampalizumab (F. Hoffmann-La Roche Ltd., Basel, Switzerland), a complement factor D inhibitor, delivered by intravitreal injection every 4 or 6 weeks [8]. In SEATTLE, emixustat hydrochloride (formerly ACU-4429, Acucela, Inc., Seattle, WA, USA), an inhibitor of the visual cycle isomerhydrolase RPE65, was administered orally once daily at 2.5 mg, 5 mg or 10 mg doses [9]. Finally, in GATE AL-8309B (tandospirone; Alcon, Fort Worth, TX, USA), a neuroprotective 5-HT1A agonist, was topically administered twice daily at concentrations of 1.0% or 1.75% [10]. All trials compared these drugs to sham treatment.

## 2. Design

### 2.1. Primary, Secondary and Composite Endpoints

The primary endpoint is the main result measured at the end of the study. A positive result in the primary endpoint is a prerequisite to accept the new intervention as effective, and it is also used as the reference for sample size calculations. Secondary endpoints evaluate additional effects of therapy. Composite endpoints combine two or more outcomes in a single endpoint to increase the number of events and decrease sample size requirements, trial length or costs.

All included phase 3 trials used the difference in the anatomical outcome between study arms, GA growth, as the primary endpoint. Fundus autofluorescence (FAF) was the imaging method used in all studies to measure the area of atrophy, but in future it may be replaced by optical coherence tomography (OCT), because this provides additional information [11], like outer nuclear layer thickness and early fluid detection. Of note, GA is a surrogate endpoint, a measurement used as a substitute of a clinically meaningful endpoint that measures directly how a patient feels, functions or survives [12]. Nonetheless, it has already been accepted by the FDA as a primary endpoint [13].

All trials used mixed-effects models to account for the repeated measurements of atrophy taken in each participant throughout the study, and all but one measured the primary endpoint in mm^2^/year (area). Instead, GATHER1 used square root transformation [14] to express the results in mm/year (linear growth), which also decreases the association between baseline area of atrophy and growth. While randomization should achieve balance between study arms in baseline area of atrophy and other potential predictors of growth (location of atrophy, multifocality, etc.), imbalances may occur by chance. To decrease this source of potential residual confounding, CHROMA/SPECTRI, DERBY/OAKS and SEATTLE included different baseline covariates in the mixed-effects models, providing an adjusted estimate of growth.

Secondary endpoints have included mainly functional outcomes, like changes in BCVA in standard and low-luminance conditions, low-luminance deficit, macular sensitivity on microperimetry, reading speed or patient-reported outcome measures (PROM) via questionnaires of quality of life related to vision [15]. Arguably, functional endpoints are more relevant to patients and are preferrred by regulatory agencies [13]. However, they have higher intra-subject variability than structural outcomes, and current tests show a slow deterioration in the typical duration of a phase 3 trial (1.5–2 years), which makes detection of changes on visual function caused by therapy difficult.

No composite endpoints have been used so far. A combination of functional endpoints may increase the sensitivity to detect visual changes in GA. Together with the development of new functional tests, this area deserves further research.

**Figure 2:**
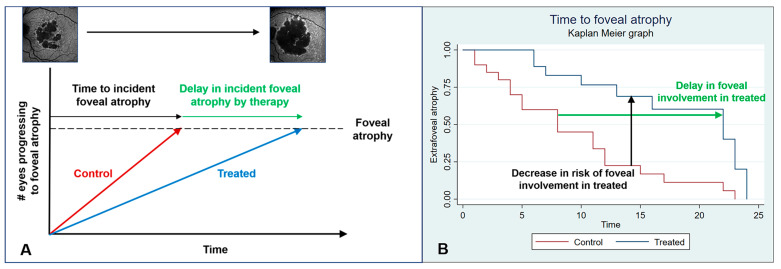
Alternative endpoints. (**A**) In extrafoveal lesions, time to, say, subfoveal atrophy should be delayed in treated (blue line) vs. untreated (red line) eyes. (**B**) From conventional Kaplan–Meier curves, one can derive the hazard ratio, the risk of progression to subfoveal atrophy by comparing treated vs. untreated eyes (vertical black line); alternatively, one can estimate the time gained without subfoveal atrophy from therapy (horitzontal green line), which is possibly a more intuitive metric.

The time to a given untoward outcome is common in other fields, but rare in GA trials (Figure 2A). These endpoints are usually analyzed with time-to-event modeling, like proportional hazards or parametric models, and plotted with Kaplan–Meier graphs (Figure 2B). The results are usually summarized as hazard ratios, which express the relative risk of experiencing the event in treated vs. control arms, given that event has not already occurred up to that point. Of note, the effect of therapy could also be expressed by the added time free of the event in the treatment arm using accelerated failure time models, [16] which may be relevant in, for example, incident subfoveal atrophy (Figure 2B). This metric is also more intuitive for the patient.

### 2.2. Eligibility Criteria

Inclusion and exclusion criteria define the set of characteristics that make patients eligible to participate in the trial. Eligibility criteria are the basis used to determine the extent to which the study results can be applied to other patients (i.e., generalizability).

The percentage of screening failures and the reasons for exclusion provide important data in this regard. The percentage of screening failure was 65% in GATHER1, 59% in CHROMA/SPECTRI and 47.5% in SEATTLE, while data are not yet available for DERBY/OAKS and were not available for GATE. When reported, the main reason for failure was ineligible GA area, suggesting that clear definitions and education on measuring the area of atrophy can minimize patient inconvenience and trial costs.

Regarding specific criteria, GATHER1 and CHROMA/SPECTRI included patients 50 years or older with GA (Table 2). This is surprising, considering that atrophy takes years to ensue, and therefore some patients could have had atrophy developing before their fifties, making alternative diagnosis more likely. In one study, more than 5% of patients with a diagnosis of dry AMD had variants in genes known to cause an inherited retinal disease when genetic testing was conducted [17], suggesting that selection of older patients is prudent to decrease the chance of mistakenly including non-GA conditions.

Careful thought should be given to inclusion of eyes with subfoveal atrophy. The advantage of excluding these eyes is that extrafoveal GA lesions progress faster [18] and the effects of therapy can therefore be established in less time and with a smaller sample. Additionally, differences in prevention of BCVA loss by incident subfoveal atrophy can presumably be established, as long as a large enough number of eyes experience this event (Figure 2). The drawbacks include a longer recruitment period and application of results to a subgroup of all patients with GA, probably less than 50% [19]. GATHER1 is the only phase 3 trial that excluded subfoveal lesions, and its results seem to support this strategy because avacincaptad pegol decreased growth by almost 30% at 12 months [6]. Similar results have been found in subgroup analyses in DERBY/OAKS with pegacetacoplan [7]. Including both locations of atrophy implicitly assumes that the same disease pathway operates in subfoveal and extrafoveal GA, which is a matter of debate [20,21].

## 3. Analysis

### 3.1. p-Value and Effect Size

The *p*-value is one of the most widely used and poorly understood statistics. It is the probability of finding a result as extreme as that obtained or greater if the null hypothesis is true. As such, it is a conditional probability: conditional on the null hypothesis being true. Although they are related, the *p*-value does not convey direct information on the effect size, which is key for medical practice. For this, one should look at absolute (difference) or even relative (ratio) metrics between trial arms and their confidence intervals (CI), which provide information regarding the likely magnitude of the effect of therapy.

In CHROMA/SPECTRI, SEATTLE and GATE, the mean progression of GA was the same or slightly larger in the treated group, and therefore *p*-values were very high or not reported at all. CHROMA/SPECTRI pre-specified a subgroup analysis in carriers of the complement factor I (CFI) risk allele, based on results of the small (*n* = 129) phase 2 MAHALO [22]. In MAHALO, CFI was one of four variants in the complement (with CFH, C2/CFB and C3) preselected for subgroup analyses. The researchers found a 49% faster growth in CFI carriers than in noncarriers in the sham group, and a 44% reduction in GA growth in CFI+ patients treated with lampalizumab as compared to sham. However, in the phase 3 trials, CHROMA/SPECTRI treatment did not slow GA progression, either overall or in CFI carriers [8]. Chance may explain the MAHALO findings, since the faster progression in CFI carriers could not be replicated in other studies [23], its biological basis is uncertain, and the analyses were not adjusted for multiple comparisons. Additionally, 26.4% of treated eyes (23/87) discontinued the study, and the main imputation method used to complete the missing data (the last observation carried forward) can induce bias [24]. Those results underscore the limitations of subgroup analyses in small samples.

On the other hand, GATHER1 and DERBY/OAKS reported positive results. As of April 2023, only pegcetacoplan has been FDA approved for GA, because results of the second, pivotal, trials required for approval of avacincaptad pegol, GATHER2, are not available yet. In GATHER1, treatment reduced progression of GA at 12 months from 0.40 mm (sham) to 0.29 mm (avacincaptad pegol 2 mg), a 27.4% reduction that was statistically significant at a *p*-value = 0.0072. The growth in the control arm was similar to that reported in untreated extrafoveal lesions in other studies [25], the effect was clinically relevant, the *p*-value was well below 0.05, and similar results were observed with the 4 mg dose (with no apparent dose–response relationship), which together provide convincing evidence of a real effect of therapy.

The 12-month results of DERBY/OAKS were difficult to interpret. OAKS revealed a reduction in growth compared to sham of 22% in the monthly and 16% in the EOM arms that was statistically significant (*p* = 0.0003 and 0.0052, respectively). However, DERBY lacked statistical significance, with a reduction of 12% in the monthly (*p* = 0.0528) and 11% in the EOM (*p* = 0.0750) arms [7]. How to proceed when the result of one of the pivotal trials is positive and the other is not? The results of both studies suggested a positive effect of the drug on GA progression, a dose–response relationship and the prespecified combined analysis showed a 17% reduction in the monthly (*p* < 0.0001) and a 14% reduction in the EOM (*p* = 0.0012) arms, with *p*-values well below 0.05. Additionally, growth was slower in the treated than in the untreated fellow eyes in the same patients (while growth in the sham treated eyes was similar to that of untreated fellow eyes in the same patients), and the results were in line with those from the phase 2 trial FILLY [26]. This supports a real effect of therapy on growth despite borderline results in OAKS and highlights the importance of looking at the whole evidence when results of a particular study seem inconclusive, which will be also discussed later on.

A related but different matter is effect size, used to assess if the difference is clinically meaningful. The *p*-value says if the effect of the therapy is probably real, while effect size helps to determine if it matters to patients. The point estimates (mean effects) of C3 or C5 inhibitors on GA progression at 12 months range from approximately −10% to −30%, depending on the drug, frequency of administration, GA location, etc. They represent the most likely value, but 95% CI provides a range of likely values in which the true effect may lie. Narrow 95% CI are desirable because they provide more precise estimates of the effect. For example, GATHER1 reported a mean difference between avacincaptad pegol 4 mg and sham of 0.124 mm/year with 95% CI between 0.038 and 0.209. These 95% CI were wide, reflecting the uncertainty caused by the relatively small number of patients. The drug effect on decreasing GA progression could be as small as 0.038 mm/year or as large as 0.209 mm/year, and it is probably real (*p* = 0.0072): if the drug is truly no different from sham, the chance of finding this difference is 0.72%.

The standardized mean difference (SMD) is used for measuring effect size in a common scale. It is the difference in mean change between treated and placebo arms divided by the pooled standard deviation [27]. SMD of 0.2, 0.5 and 0.8 are regarded as small, medium and large, respectively. Using the published results in GATHER1, the SMD of avacincaptad pegol vs. sham at 12 months was 0.15 (2 mg) and 0.18 (4 mg). Other metrics have been discussed elsewhere [27].

### 3.2. Study Power/Sample Size

The power of a study is the probability of showing a difference between treatments when it truly exists. For a given level of significance or false positive rate (typically set at a two-sided α = 0.05 or 5% in superiority trials), power increases when the difference in the primary endpoint between study arms increases, when the variability of the primary endpoint is low and when sample size is large. In addition, an allocation ratio (the distribution of trial participants to treatment or control arms) of 1:1 requires a smaller sample than other ratios (2:1, 3:1, etc.), but this comes at the expense of being less attractive to patients in placebo-controlled trials, and a decreased ability to detect adverse events of the new therapy.

A summary of the assumptions used to calculate the sample size is provided in Table 3. Assumptions for sample size calculations in GATHER1 with avacincaptad pegol were derived from the phase 2 trial FILLY with pegcetacoplan [6]. Sample size assumptions from DERBY/OAKS were not yet available, and GATE did not report them in its main publication. The total sample size ranged from 286 in GATHER1 to 975 in SPECTRI, a ratio of 3.4. The estimated annual growth rates in the control group differed modestly between trials, but effect sizes with reductions of 20–30% of GA growth and estimated yearly discontinuations near 15% were common.

### 3.3. Intention-to-Treat (ITT) Principle

The ITT analysis specifies that patients (or eyes) are analyzed in the treatment group in which they were originally randomized, regardless of the treatment they eventually received [28]. That means that if someone was randomized to receive an intravitreal injection, but they received the sham procedure, they will be analyzed in the active treatment group. Simply stated, it is the indication or intention to treat a subject with a given treatment (defined by randomization) that matters. This is the standard approach to data analysis in phase 3 trials, because it includes all patients, preserves the benefits of randomization (balancing known and unknown factors that may affect response to treatment between groups), and provides a conservative estimate of the effect of treatment, which is closer to what would be observed if it were administered to the population as a whole. The *per-protocol* (PP) analysis population includes only cases fully adherent to the protocol [28]; as such, PP shows the maximal effect of therapy, i.e., under optimal compliance. Finally, the *as-treated* population analyzes patients according to treatment actually received [28]. Unfortunately, PP and *as-treated* analyses can induce selection bias and confounding.

Except for GALE, where this information was not provided, all trials acknowledged following the ITT principle. In some trials, a *modified* ITT analysis was used, meaning that patients were included in the ITT analysis if they met certain prerequisites, like having received at least one intravitreal injection and having a post-treatment visit available. However, a modified ITT may mean different things in different trials, and the reader must pay attention to how it is defined. In SEATTLE and GALE treatment was self-administered for 24 months. In this scenario, incomplete adherence to treatment or crossovers are a concern, and ITT becomes important to minimize bias. In future trials with self-administered therapies, it would be important to monitor non-compliance and compare the results from ITT and PP analyses. In the other trials, therapy was administered by medical personnel, and thus compliance is known, although non-adherence, crossovers and missing data could also compromise results.

### 3.4. Missing Data

Pivotal phase 3 trials in ophthalmology usually follow several hundred patients for 18–24 months. Therefore, missing data is unavoidable. This has two consequences: (1) loss of power (in superiority trials, it is more difficult to show a beneficial effect of therapy, even if it truly exists); and (2) biased results if those who discontinue are different from those who remain in the study or if they withdraw for reasons related to the therapy. Keeping a high visit rate is a challenge, particularly for studies ongoing in the 2020–2022 period (SAGA, GATHER1/2 and DERBY/OAKS) due to the COVID-19 pandemic. Minimizing missing data during the study is paramount, and when this occurs, several statistical methods can be used to impute values and try to decrease its impact. These range from the simple last-observation-carried-forward, in which a missing follow-up value is replaced by the previously observed value, to sophisticated multiple imputation procedures, where an average of the imputed values from multiple imputed datasets is estimated, while acknowledging the uncertainties in the estimated value.

Important visits at which every effort should be made to collect all data are baseline, the visit where the primary endpoint is measured, and the end-of-study visit. Missing data can also affect exams within a visit; for example, a missing FAF image. Discontinuation was very variable between studies and ranged from 7.9% in CHROMA/SPECTRI to 37.0% in SEATTLE. In SEATTLE, the main reason for this was incident adverse events, which generally increased with increasing drug dose. In DERBY/OAKS, 11.4% of patients discontinued the study before month 12, while the percent of missed injections was 11.9% (roughly half of them for COVID-19-related reasons). On the other hand, 29.9% of participants in GALE did not finish the study, but the percentage was similar between study arms. Even in this situation, bias can occur because patients leaving the study may be different to those who remain for reasons related to the treatment. Note that patients who discontinue are included in the ITT/modified ITT analyses, sometimes using imputation methods, and sensitivity analyses may be used to evaluate the robustness of the results under different assumptions [22].

### 3.5. Consistency of Results

Although for regulatory purposes, the key results are those from pivotal phase 3 trials, all available evidence should be taken into account to determine the effects of a given therapy. This includes data from phase 2 trials or, rarely, external evidence in cases in which the therapy is already approved for another indication and tested for the condition of interest. Some of the methods that can be used to quantitatively integrate this information include meta-analyses and Bayesian approaches.

**Figure 3:**
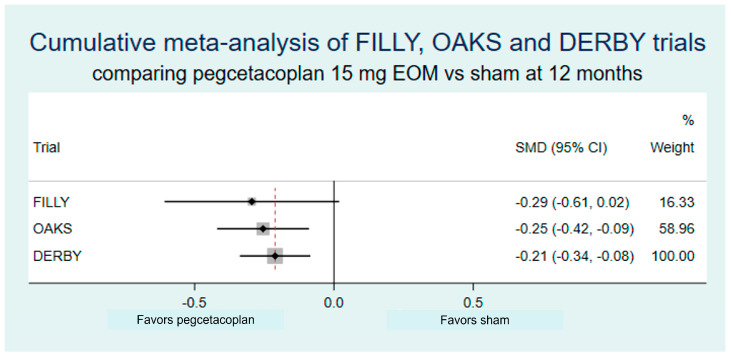
Estimated cumulative meta-analysis of the effects of pegcetacoplan 15 mg every other month on the growth of geographic atrophy at 12 months in the FILLY (phase 2), OAKS and DERBY (phases 3) clinical trials. A modest but real effect is shown when considering all results. CI: confidence interval; SMD: standardized mean difference.

A meta-analysis combines the results of many studies on the same disease and endpoint to provide the most precise estimate of the effect of therapy [29]. Essentially, it is a weighted average of the effects of therapy across studies. A cumulative meta-analysis performs successive meta-analyses, one each time a new study is added, which makes it possible to determine the effect of the most recent study on the overall estimate. This would have been useful in the case of pegcetacoplan, where the primary endpoint results in OAKS were statistically significant, but those of DERBY were not. An estimated cumulative meta-analysis of the phase 2 FILLY [26], and the phase 3 OAKS and DERBY shows that, while the results of the latter attenuated the benefits of the drug, they remained statistically significant (Figure 3).

Bayesian methods [30] are less well known and are rarely used in ophthalmology trials. These approaches require that one expresses a plausible distribution of the effects of therapy before the current study based on what is known up to that point (the so-called *prior*), for example the results of a phase 2 trial. Then, one uses Bayes’s theorem to update that prior belief with data from recent evidence (the phase 3 trials) to provide a posterior distribution, or a range of likely values were the true effect of therapy lies.

## 4. Interpretation

### 4.1. Efficacy–Safety Balance

Recent episodes of intraocular inflammation with an otherwise effective antiangiogenic drug in patients with exudative AMD (eAMD) [31] underscore the importance of evaluating both efficacy and safety to establish the effects of an intervention. Table 4 shows a summary of the most commonly observed side effects in the active arm of published GA phase 3 trials. It should be noted, however, that these events were not necessarily more frequent in treated than in placebo arms. We focus the rest of this section on studies in which at least one trial has reported positive results.

The combined results of OAKS and DERBY at 24 months showed an increased rate of new-onset eAMD in treated eyes. There was a dose–response relationship that roughly doubled the risk of eAMD from sham to EOM and from EOM to monthly dosing: 3.1%, 6.7% and 12.2%, respectively. The rates were slightly lower in patients without fellow eye eAMD. The percentage of eAMD cases in the avacincaptad pegol arm was also larger than the sham-treated group in the small GATHER1 trial, suggesting that these drugs do increase exudative events. Cumulative events of intraocular inflammation at 24 months were 2.1% in EOM and 3.8% with monthly dosing, with no reports of occlusive or nonocclusive vasculitis. The rate of endophthalmitis was approximately 1:3000 injections (similar to that reported with conventional antiangiogenic therapy) [32], which is reassuring, considering that these therapies downregulate the innate immune system. Simultaneous treatment of GA with pegcetacoplan and eAMD with antiangiogenic therapy was allowed in DERBY and OAKS, and preliminary results seem to suggest that combined therapy is safe and efficacious. Nonetheless, the increased risk of short-term decrease in BCVA from eAMD must be discussed with the patient when considering treatment with pegcetacoplan for the prevention of long-term vision loss.

### 4.2. Application of Results to My Patients

It is essential to determine whether the results of the trial can be applied to my patients, because the intervention may be influenced by demographic, biological or socioeconomic factors. A good place to start is reviewing “Table 1” of the trial manuscript, which will usually describe the characteristics of the participants. Other considerations also apply, some of which are discussed here in the context of pegcetacoplan, the only approved therapy as of 11 April 2023.

Since many conditions mimic GA secondary to AMD [33], a first step is confirming that the patient does indeed present this disease. Many disorders can cause macular RPE atrophy, but in none of them has C3 upregulation been demonstrated to play a pathogenic role, and the effect of pegcetacoplan is unknown in these cases. The efficacy and safety of the drug in patients below 60, in non-Caucasians (who represented less than 10% of participants in the phase 3 trials) and with different complement-related genetic variants need to be studied and considered carefully. Results were better in extrafoveal than subfoveal lesions, although the *p*-values for interaction between treatment and atrophy location are not known by the authors. In terms of safety, the diagnosis in the fellow eye may play a role on the risk of incident eAMD in pegcetacoplan-treated eyes, and the added burden of compliance with two intravitreal drugs may jeopardize results.

Another issue to consider will be adherence to treatment, which is a common reason of poor translation of results from clinical trials [34]. This will depend on both patients and practitioners. The costs of therapy and the organization of healthcare services, public and private, will be determinants of adherence. The open-label extension GALE (NCT04770545), phase 4 and real-world evidence studies will inform the impact of compliance on drug effectiveness.

## 5. Discussion

Approval of medical therapies is based on sound, rigorous clinical trials. We reviewed some points related to their design, analysis and interpretation, and additional information on similar topics can be found elsewhere [35,36].

Overall, some of the trials surveyed did not report information on important aspects of the study. Adherence to the CONSORT reporting guidelines [37] will improve communicating of what was done, allowing a better interpretation of the results.

As can be seen in Table 5, there were differences between trials in terms of the population included, the analytical approach, the quantity of missing data at the end of the study or the type of adverse events. This is not unexpected, since the design and analysis of the trials are adapted to the nature of the intervention. However, key aspects in the interpretation of any study include the need to assess the application of the results to the particular patient and the judicious evaluation of both efficacy and safety.

Readers should also bear in mind that small studies have less precision and wider confidence intervals (Figure 3), and thus can only report statistically significant results if the effect size is very large. When combined with publication bias (the trend of publishing studies with positive results), published small trials tend to overestimate the effects of therapy [38]. This overestimation of effect occurred, for example, in the lampalizumab and pegcetacoplan trials.

Geographic atrophy is a notable exception in research in macular diseases, inasmuch as the primary endpoint in all phase 3 clinical trials conducted thus far has been based on an anatomic change, which is a surrogate endpoint. In the case of the DERBY/OAKS trials, the positive effect of pegcetacoplan on the growth of area of atrophy was observed a few months after starting treatment, while its beneficial effect on visual function may take a few years. In addition, this therapy can increase the risk of short-term visual acuity loss due to incident eAMD. This is a key issue, considering that patients with this condition are elderly subjects with a relatively short life expectancy, estimated as 6.4 years in one study [39]. Adherence to therapy will also play an important role and will be influenced (among other things) by the cost of therapy, which may be close to 2000 USD/injection. All things considered, the benefits of therapy from the patient’s perspective are, at least, debatable. In this context, PROMs may provide critical information, while economic evaluation through cost-utility (estimation of cost-per-QALY (quality-adjusted life year, one year lived in perfect health)) and cost-effectiveness analysis (comparison of costs and outcomes with and without treatment) will provide a useful perspective of the effects of this intervention for all those involved.

In summary, we reviewed some methodological aspects of phase 3 clinical trials. When applied to GA, similarities and differences between studies were noted. Poorly reported topics were highlighted, paving the way for improvement in future studies.

## Figures and Tables

**Table 1:** Topics discussed.

Design	Analysis	Interpretation
Primary, secondary, and composite endpoints Eligibility criteria	*p*-value and effect size Study power/sample size Intention to treat and treatment adherence Missing data Consistency of results	Efficacy–safety balance Application of results to my patients

**Table 2:** Summary of phase 2/3 or 3 clinical trials in GA secondary to age-related macular degeneration.

Trial	Sponsor	Patients (Total *n*)	Drug, Route	MoA	Primary Endpoint	Results
SAGA, Sep/23	Alkeus	≥60 years At least one eye with GA (ongoing; planned *n* = 300)	ALK-001, oral	Slowing vitamin A dimerization	Difference in growth rate	Study ongoing
GATHER1/2, [6] Jul/23	IVERIC bio	≥50 years Non-foveal GA, but atrophy ≤ 1500 µm from foveal center (*n* = 286)	Avacincaptad pegol (Zimura^®^), IVI	Complement regulation (anti-C5)	Difference in growth rate at 12 months after the square root transformation *	(% reduction in treated vs. sham) 2 mg: 27.4% (*p* = 0.0072) 4 mg: 27.8% (*p* = 0.0051)
DERBY-OAKS, [7] Jan/23	Apellis	≥60 years GA area ≥ 2.5 and ≤17.5 mm^2^ (*n* = 1.258)	Pegcetacoplan (Syfovre^®^), IVI	Complement regulation (anti-C3)	Difference in growth rate at 12 months	(% reduction in treated vs. sham) DERBY: Monthly: 12% (*p* = 0.0528) EOM: 11% (0.0750) OAKS: Monthly: 22% (*p* = 0.0003) EOM: 16% (*p* = 0.0052)
TOGA, Nov/20	MEDARVA Foundation	≥55 years GA ≥ 0.5 and ≤7 disc areas (*n* = 286)	Doxycycline (Oracea^®^), oral	Antiinflammatory	Difference in growth rate at months 6 and 30	Not published
CHROMA-SPECTRI, [8] Jan/18	Roche	≥50 years Well demarcated GA area (*n* = 1.881)	Lampalizumab, IVI	Complement regulation (anti-Factor D)	Difference in mean change in GA area at week 48	(lampalizumab minus sham) CHROMA: q4w: −0.02 mm^2^ (*p* = 0.80), q6w: +0.05 mm^2^ (*p* = 0.59); SPECTRI: q4w: +0.16 mm^2^ (*p* = 0.048 favoring sham), q6w: +0.09 mm^2^ (*p* = 0.27)
SEATTLE, [9] May/16	Acucela Inc.	≥55 years Clinical diagnosis of GA (*n* = 508)	Emixustat hydrochloride (ACU-4429), oral	Visual cycle modulation (anti-RPE65)	Difference in growth rate over 24 months	Treated: 1.69 to 1.84 mm^2^/y Sham-treated: 1.69 mm^2^/y (*p* ≥ 0.81)
GATE, [10] May/12	Alcon	≥55 years GA area ≥ 1.25 and ≤20 mm^2^ (*n* = 768)	AL-8309B (tandospirone), topical ocular	Neuroprotective (5-HT1A agonist)	Difference in mean annualized lesion growth rate up to 30 months	AL-8309B 1.0%: 1.73 mm^2^/y AL-8309B 1.75%: 1.76 mm^2^/y Vehicle: 1.71 mm^2^/y

Date under trial name is its estimated study completion date. Available on https://clinicaltrials.gov/, accessed on 20 May 2023. BCVA: best-corrected visual acuity; EOM: every other month; GA: geographic atrophy; IVI: intravitreal injection; MoA: mechanism of action; q4w: treatment every 4 weeks; q6w: treatment every 6 weeks; y: year. * This is based on subtracting the square root of the area of atrophy at the end of study from the square root of the area of atrophy at baseline divided by the time between visits in years to determine GA growth.

**Table 3:** Summary of assumptions used to calculate sample size.

	GATHER1	CHROMA/SPECTRI *	SEATTLE
Power (1-β), %	90	88	80
Approximate α (unadjusted)	0.025 **	0.0495	0.05
Growth in the control group (SD), mm^2^/y	0.33 (0.20) ***	2.08 (1.53)	1.75 (1.20)
Difference between trial arms (SD), mm^2^/y	0.10 (0.20) ***	0.42 (1.53)	0.56 (1.20)
Randomization ratio	1:2:2	2:1:2:1	1:1:1:1
Expected yearly drop-out rate, %	15–20	15	16.7

* Overall patient population; ** one-sided test; *** mm/year; SD, standard deviation.

**Table 4:** List of the most common adverse events reported in published phase 3 clinical trials in geographic atrophy in the study treatment arms of each study. For trials with more than one active treatment arm, the results from the two dosing regimens or drug concentrations are pooled.

	GATHER1	DERBY/OAKS *	CHROMA/SPECTRI	SEATTLE	GATE
Ocular	Conj. hemo. (24.7%) eAMD (9.3%) Increased IOP (6.7%)	eAMD (9.4%) ** IOI (3%) Endophthalmitis (1.2%)	Conj. hemo. (29.6%) Increased IOP (11.3%) Eye pain (10%)	Delayed DA (55.4%) Chromatopsia (17.6%) Visual impairment (15.4%)	Reduced VA (32.1%) Eye irritation (9.1%) Cataract (8.1%)
Non-ocular	UTI (9.3%) Fall (6.7%) Nasoph. (6%)	Not reported	URTI (8.6%) Fall 8.6%) Bronchitis (5.5%)	Fall (9.7%) Nasoph. (9.2%) Hypertension (9.6%)	Not reported

Conj. hemo.: conjunctival hemorrhage; DA: dark adaptation; eAMD: exudative age-related macular degeneration; IOI: intraocular inflammation; IOP: intraocular pressure; nasoph: nasopharyngitis; URTI: upper respiratory tract infection; UTI: urinary tract infection; VA: visual acuity. * Pending detailed reporting; ** As determined by the investigator.

**Table 5:** Summary of some of the topics discussed.

	GATHER1	DERBY/OAKS	CHROMA/SPECTRI	SEATTLE	GATE
Metric of PE	mm/year	mm^2^/year	mm^2^/year	mm^2^/year	mm^2^/year
Adjusted PE	No	Yes	Yes	Yes	No
Atrophy location	Extrafoveal	Any	Any	Any	Any
Intention to treat	Yes	Yes	Yes	Yes	No?
Discontinuation, %	20.1	11.4	7.9	37.0	29.9
Specific adverse events	eAMD	eAMD	Increased IOP	Delayed DA	Reduced VA

DA: dark adaptation; eAMD: exudative age-related macular degeneration; IOP: intraocular pressure; PE: primary endpoint; VA: best-corrected visual acuity.

## Data Availability

Not applicable.
